# Construction and Validation of a Measurement Instrument for Attitudes towards Teamwork

**DOI:** 10.3389/fpsyg.2017.01009

**Published:** 2017-06-20

**Authors:** Santiago Mendo-Lázaro, María I. Polo-del-Río, Damián Iglesias-Gallego, Elena Felipe-Castaño, Benito León-del-Barco

**Affiliations:** ^1^Department of Psychology and Anthropology, University of ExtremaduraCáceres, Spain; ^2^Department of Didactics of Music, Plastic and Body Expression, University of ExtremaduraCáceres, Spain

**Keywords:** attitudes, teamwork, university students, learning, academic goals

## Abstract

Cooperative, collaborative learning and other forms of group learning methods are increasingly used in classrooms. Knowing students’ attitudes toward teamwork has great value since they influence the students’ learning results as well as their social development. So it is necessary to have robust instruments to provide a better understanding of these attitudes and preferences concerning teamwork. Such instruments also help to identify the factors that promote positive or negative attitudes within the context of group activities. Using a sample of 750 first and second year university students studying a degree in Kindergarten, Primary and Social Education, an instrument measuring attitudes toward team learning has been developed. Two distinct factors were obtained through various factorial analyses and structural equations: *Academic attitudes* and *Social and emotional attitudes*. Our study reveals that the instrument is both valid and reliable. Its application is both simple and fast and it has important implications for planning teaching and learning activities that contribute to an improvement in attitudes as well as the practice of teaching in the context of learning through teamwork.

## Introduction

Methods of cooperative and collaborative learning and other forms of group learning are increasingly being used in the classroom to promote teamwork among students, improve performance and learning, and develop interpersonal competences (Venter and Blignaut, 1998; Johnson et al., 2007; Gottschall and García-Bayonas, 2008; Gaudet et al., 2010; Kirschner et al., 2011; Mendo et al., 2016); or to internalize the values, attitudes and patterns of behavior needed to turn students into involved and contributing citizens in a democracy (Johnson and Johnson, 2016).

However, in spite of its advantages, teamwork is not always received positively by students (Burdett, 2006; Payne and Monk-Turner, 2006; Hammar Chiriac, 2014). It is not enough to assign students a task and tell them to work together. The following aspects are essential to introducing teamwork in the classroom: the teacher’s preparation in the use of methodologies favoring peer cooperation, students’ training in teamwork, the team’s social skills (Rodríguez and Ridao, 2014; León et al., 2015), assessment (Beigi and Shirmohammadi, 2012), team composition (Hijzen et al., 2007), task design (Nokes-Malach et al., 2015) and the team’s beliefs in its efficacy and performance, interdependence, group climate, cohesion and conflicts (León et al., 2017).

When these elements are not taken into account, unsatisfactory work experiences may discourage people from becoming involved in teamwork. Such negative experiences can lead to negative attitudes toward teamwork, which can affect their future teamwork experiences. In contrast, a positive learning experience can improve attitudes toward teamwork, efficiency and cooperation with classmates, which would improve students’ willingness to work as a team in class (Ekimova and Kokurin, 2015).

In this sense, students’ preference for teaching methods is one of the factors that affects the teaching/learning process (Anderton-Lewis and King, 1995). Thus, for teamwork to be effective, the team members must perceive it as an attractive way to work (Lembke and Wilson, 1998). So, when students are not well disposed to teamwork, or they are not very willing to participate for whatever reasons, it is very difficult to achieve the objectives of learning and social and interpersonal development through the different teamworking methods.

One of the most classic definitions of attitude is that proposed by Allport (1935), who considered it “a state of mental and neural disposition organized by means of experience, which exerts a directive or dynamic influence on the individual’s response to all kinds of objects and situations” (p. 810). According to Gardner and Korth (1998), attitude toward teamwork is defined as the individual will (internal state) to continue working with the same team, as well as with other teams (personal action).

The structure of attitudes is mainly represented by three, two, or one-dimensional models. The three-dimensional model includes three attitudinal components: (a) cognitive, (b) affective, and (c) behavioral. According to the two-dimensional model, attitude consists of affective and cognitive components. The unidimensional model emphasizes the evaluation of the attitudinal object in terms of positive–negative; sympathy–antipathy; approach–rejection (Ubillos et al., 2004).

According to Lobato (1998), the goals of teamwork are of an intellectual (conceptual learning, creative problem solving, intellectual skills…) and social (interpersonal relations, attitudes toward classmates…) nature, in which contents, procedures, attitudes and skills are particularly relevant.

A positive attitude toward teamwork is essential; it is one of the mechanisms involved in a team’s positive, academic and social outcomes and can only be developed if a competitive individualistic orientation is set aside (Castelló, 1998). This implies abandoning the belief that success depends only on one’s own effort and requires trust in the capacity of the team members.

The success of learning is determined by the positive beliefs that students have concerning teamwork (Hijzen et al., 2006). Students’ different attitudes to teamwork as a result of past experiences may make decision-taking, cooperation and coordination between team members more difficult (Fransen et al., 2013). Nevertheless, positive attitudes on the part of certain team members may help to soften the attitudes of the least positive members (Ekimova and Kokurin, 2015).

Although the variables that condition the effectiveness of teamwork by university students are numerous, and it is difficult to determine the specific influence of each one, we start from the idea that students’ attitudes toward teamwork is one of the fundamental variables influencing their social development and learning results. So, determining how students perceive and value teamwork is essential (Lobato, 1998; Johnson et al., 1999; Mena et al., 2013; Mujika et al., 2013).

Just as students’ attitudes toward the subject matter are taken into account so as to avoid groups in which negative attitudes predominate (Serrano and Calvo, 1994); it is also important to pay attention to the attitudes shown toward teamwork, as an differentiating element of participation, involvement, interest, satisfaction or confidence in the group.

From this arises the need for instruments that can bring a better understanding of the attitudes and preferences concerning teamwork, which can help to identify the factors that encourage a positive or negative attitude in the context of group activities, as well as to examine the situations students are subjected to.

Research on students working in teams (although scarce) shows contradictory results. On the one hand, when the students’ goal is to achieve a good performance, most prefer individual work, but on the other hand, they do acknowledge the need for teamwork to improve interpersonal skills (McCorkle et al., 1999; Ruiz Ulloa and Adams, 2004).

There are many factors involved in the formation of attitudes toward teamwork in university students. Pfaff and Huddleston (2003) found that the perceived workload, the time spent in class, the use of peer assessment, and the absence of ‘free riders’ (team members who benefit from the effort of others in the team without contributing themselves) are significant predictors of favorable attitudes toward teamwork. Hall and Buzwell (2012) found that free-riders is the factor that causes university students the most concern. Likewise, if, during the process of teamwork, there is mature communication, responsible interdependence, psychological security, a common purpose, clear roles and goals, then the experience will have a positive effect on individuals’ attitudes toward teamwork (Ruiz Ulloa and Adams, 2004). Recent studies have found that concerns about the results of the assessment of teamwork and perceptions of the teamwork environment affect students’ attitudes toward teamwork (Beigi and Shirmohammadi, 2012). Similarly, Ekimova and Kokurin (2015) found that the qualification received by the team is the most significant predictor of students’ attitudes toward teamwork.

In an exploratory study of attitudes toward teamwork of first year engineering students, Alford et al. (2014) identified three elements that influence students’ perception of teamwork (fun, frustration and learning): (a) if the task is interesting and challenging, but feasible, the students have fun; (b) if there is clear communication, confidence in the abilities of others, and understanding of differences and commitment, frustration is reduced; (c) the task and the students’ general attitude toward teamwork influences their perception of learning.

On the other hand, Urdan and Maehr (1995) or Anderman and Anderman (1999) among others, point out that social goals must be taken into account in the study of motivation, because students may have social reasons for their performance and behavior. Accordingly, there have been different attempts to integrate the study of goals and motivation, given that motivation influences the meaning or valuation of an activity and how it is dealt with (Alonso-Tapia, 2005). When students face a learning situation, they propose the desired goals and the necessary strategies and resources (Valle et al., 2000), i.e., their individual reasons or purpose of the activity.

Thus, as with attitudes toward teamwork, learning goals determine the way we face and respond to learning situations in a group. In addition, teamwork influences a student’s individual motivation (Krishen, 2013) and his/her learning goals. This is because working in a team increases the perception of competence and control over the activity, thus increasing the enjoyment of the task (León et al., 2011). This interaction between attitudes toward group learning and academic goals shows the pertinence of their joint analysis.

### The Present Study

However, several different investigations have focused on the assessment, using different instruments, of some of the variables related to attitudes toward teamwork; such as the preference or appraisal of the teamwork experience (Pfaff and Huddleston, 2003; Gottschall and García-Bayonas, 2008; Alford et al., 2014; Rudawska, 2017), motivation (Ibarra and Rodríguez, 2007; Järvelä et al., 2010), assessment and work environment (Beigi and Shirmohammadi, 2012), the team’s potency (León et al., 2017), the quality of the product and process, classmates’ support, or interdependence and frustration (Nausheen et al., 2013). We believe it is necessary to have instruments that can contemplate these and other variables identified in the previous research involved in attitudes toward teamwork, such as: interest in the task and motivation, the time dedicated to it, the learning and decision-taking, or the interpersonal relations.

Thus, the aim of this study is to build an instrument that can contribute to the understanding, and permit the evaluation, of attitudes toward teamwork in the university context, taking two great dimensions (the academic and the social) as the starting point, as these are linked to both the educational process and the aims of teamwork, grouping together and synthesizing the main variables involved in the formation of attitudes toward teamwork. The availability of an instrument to evaluate attitudes toward teamwork will provide teachers with information that can help them plan, intervene in and evaluate the teamwork process.

## Materials and Methods

### Participants

In this work, the participants were 750 students (71.4% females and 28.6% males) aged between 18 and 36 years. The mean age is 20.62 years (*SD* = 2.45). The participants were first and second year students of the undergraduate degrees (edited to ensure anonymity) in Child Education, Primary Education, and Social Education.

We chose students from these degrees due to the large quantity of assessable contents and activities related to teamwork that these students must carry out from the first years of their university training, ensuring that the participants in the study had had contact with teamwork in the university setting.

### Instruments

An *ad hoc questionnaire* was used to collect information concerning age, gender, degree course and year, as well as the preference for working alone or in a team. To determine this preference, we asked students to choose between: 1 (*I prefer working in a team*); 2 (*I don’t care whether I work alone or in a team*); 3 (*I prefer working alone*).

*“Cuestionario de actitudes hacia el trabajo en equipos de aprendizaje”* (CACTE, Questionnaire on Attitudes toward Learning Teams). There are traditionally accepted measurement procedures based on the fact that attitude is a latent construct; i.e., attitudes can be inferred from people’s behavior or opinions. Hence the appropriateness of using scalar methods that provide information about the degree or intensity of an attitude toward its object, with self-reporting measures being predominant (Ubillos et al., 2004).

A prior review of the available literature on instruments used to measure attitudes toward teamwork did not identify a specific instrument with adequate psychometric characteristics that allowed the main variables identified in the formation of attitudes toward teamwork to be measured simply and clearly.

So, following the recommendations of Vallejo (2006) on the construction of scales to measure attitudes in psychology and education, the CACTE was developed, taking as its starting point the surveyees’ responses as a function of their ideas, feelings, beliefs, etc., on a 5-point Likert-type scale. They rated their degree of agreement from 1 (*completely disagree*) to 5 (*completely agree*) on two dimensions (*academic* and *social affective)* that influence students’ appraisal of learning teams. The academic dimension refers to the actions, beliefs, appraisal and valuation of teamwork as a function of expectations about the outcome of their learning and individual success. The social dimension is related to their appraisal of the interaction when working with others. Together, these two dimensions form the attitude toward learning teams in terms of a positive or favorable appraisal and a negative or unfavorable one.

*Cuestionario de Potencia de Equipos de Aprendizaje (CPEA)* [Learning Team Potency Questionnaire]; (León et al., 2017). The CPEA assesses students’ perception of their work team’s capacity to successfully perform the activities in the different subjects. It is made up of 8 Likert-type items with ten response options ranging from 1 (*completely disagree*) to 10 (*completely agree*). The CPEA has two factors: the first, *Confidence* (4 items), assesses students’ expectations about their own team’s efficacy. The second, *Performance* (4 items), assesses students’ perception of their team’s capacity to successfully perform a series of academic tasks. Example items are: F1: “It is easy for my team to carry out any activity proposed in the different subjects”; F2: “The teamwork carried out by my team is of a very high quality.” The alpha indexes (α = 0.91), composite reliability (CR = 0.93) and McDonald Omega (Ω = 0.92) show that the CPEA presents good global reliability and average extracted variance (AVE = 0.65). The two factors of the questionnaire present adequate reliability and an AVE > 0.50 in both factors [F1 (α = 0.88, CR = 0.88, Ω = 0.85, AVE = 0.59); F2 (α = 0.83, CR = 0.80, Ω = 0.82, AVE = 0.51).

The *Achievement Goal Questionnaire* (AGQ) of Hayamizu et al. (1989), adapted by Hayamizu and Weiner (1991), translated into Spanish. This questionnaire consists of 20 statements about a student’s reasons for studying. Responses are rated on a Likert-type scale ranging from 1 (*never*) to 5 (*always*). It analyzes three goal orientations: (a) Learning Goals (LG; 8 items) assesses the students’ tendency to engage in academic tasks with the goal of learning, acquiring new knowledge and increasing their competence; (b) Achievement Goals (AG; 6 items) reflects the students’ tendency to learn in order get good grades in the exams and to advance in their studies; and (c) Social Reinforcement Goals (SRG; 6 items) analyzes the students’ tendency to learn in order to gain approval and avoid rejection by parents and teachers. The questionnaire has good reliability and adequate AVE in all three factors [F1 (α = 0.88, CR = 0.92, Ω = 0.85, AVE = 0.60); F2 (α = 0.83, CR = 0.85, Ω = 0.82, AVE = 0.50); F3 (α = 0.83, CR = 0.90, Ω = 0.80, AVE = 0.63)].

### Procedure

We contacted the participants (*n* = 750) in the classroom during the academic year 2015/2016. The study received approval from the Ethics Committee of the University of Extremadura. All the participants were treated in accordance with the ethical norms of the American Psychological Association as far as consent, confidentiality and anonymity of the answers were concerned. After obtaining their informed consent, they completed the CACTE, the CPEA, and the AGQ anonymously, and the confidentiality of the data and their exclusive use for research purposes was ensured. The administration took place at the beginning of each class and lasted approximately 15 min. Subsequently, in order to establish temporal reliability, 17 weeks later, 200 of the participants again completed the CACTE following the same procedure.

### Data Analysis

Initially, for the development and analysis of the psychometric characteristics of the “Questionnaire of Attitudes toward Learning Teams,” the principal components exploratory factor analysis (EFA) with varimax rotation was carried out, obtaining a two-factor solution.

After the EFA, the factor structure found was confirmed with a confirmatory factor analysis (CFA). To determine the invariance by gender of the obtained model, a multi-group analysis was performed. The stability and factor loadings of the model were established with the bootstrap method. Subsequently, correlations and comparisons of means were calculated to establish convergent and nomological validity.

The reliability of the CACTE (12 items) and of the two factors (6 items) was calculated with Cronbach’s alpha, the composite reliability coefficients, McDonald’s Omega and the AVE.

To determine the use of parametric or non-parametric tests when analyzing the existence of relations and/or differences in the scores of the CACTE, the CPEA and the AGQ, the assumptions of normality, randomization, and homoscedasticity were contrasted, concluding that the use of parametric tests was appropriate.

The EFA, correlations and comparisons of means were performed with the SPSS-21 program, and for the CFA, the AMOS-21 program was used.

## Results

### Exploratory Factor Analysis

The original sample (*n* = 750) was divided into two randomly extracted subsamples (*n*_1_ = 375 and *n*_2_ = 375). The first one (*n*_1_) was used to carry out the EFA, and the second (*n*_2_) was used as a validation sample for the CFA. Both subsamples are equivalent as regards age, *t*(748) = 0.763, *p* = 0.446, and gender, χ^2^(1) = 2.317, *p* = 0.128.

In the first EFA, the items that had corrected homogeneity indexes lower than 0.30 were eliminated (*Getting good or bad grades should only depend on my own effort, Teamwork prevents errors,* and *Teamwork causes problems with the classmates*).

The sample adequacy measurement (KMO = 0.886) and Bartlett’s sphericity test [χ^2^ = 650.203(66), *p* < 0.001] indicated that factor analysis was appropriate.

Lastly, using EFA with principal components and varimax rotation, a two-factor solution was obtained (**Table 1**), which explained 62.0% of the total variance. The first factor, *Academic attitudes* (6 items), explained 32% of the variance and reflects the appraisal of academic consequences derived from teamwork, with Items 5 and 6 inversely worded. The second factor, *Social and affective attitudes* (6 items), explained 30% of the variance and groups the appraisal of the interactions with other classmates during teamwork. These two factors have a correlation of 0.720 (*p* < 0.001).

**Table 1:** Exploratory Factor Analysis of the Questionnaire of Attitudes toward Learning Teams (CACTE).

	Items	*M*	*SD*	F1	F2	Communalities
1	Working in a team increases my interest and motivation for the topics covered	3.82	1.00	0.795	0.155	0.632
2	The quality of the work improves when performed in a group	3.82	0.88	0.775	0.249	0.611
3	My grades improve when I work in a team	3.43	0.98	0.722	0.233	0.521
4	Teamwork is important for my training	4.08	0.82	0.662	0.221	0.455
5	Teamwork seems a waste of time to me^∗^	4.26	0.95	0.635	0.194	0.403
6	I learn more when working alone than in a team^∗^	3.47	1.07	0.624	0.245	0.392
7	I feel useful and appreciated by my teammates	4.19	0.73	0.268	0.813	0.680
8	I feel comfortable working with my classmates on team activities	4.31	0.84	0.255	0.798	0.660
9	Teamwork favors friendly relations	4.19	0.84	0.242	0.696	0.503
10	I am confident that my teammates will fulfill their share of the work	4.17	0.85	0.231	0.693	0.536
11	Teamwork helps me to know my classmates better	4.53	0.53	0.193	0.684	0.476
12	Consensus among the team members helps to make better decisions	4.46	0.63	0.127	0.604	0.394

*F1 = Academic attitudes (Eigenvalue: 3.734; explained variance: 32%); F2 = Social and affective attitudes (Eigenvalue: 3.529; explained variance: 30%). ^∗^Items inversely worded and recoded.*

The internal consistency of the questionnaire, measured with a Cronbach’s alpha of 0.905, was very acceptable. Internal consistency was acceptable for the factors *Academic attitude* (α = 0.839) and *Social and affective attitude* (α = 0.869). With regard to the temporal reliability, the test–retest reliability coefficient (*r* = 0.870, *p* < 0.001) indicated a high stability of the scores.

### Confirmatory Factor Analysis

The CFA was performed with the second subsample (*n*_2_ = 315) in order to confirm the number of factors found in the EFA and determine whether or not they are related to each other or whether they are independent. The analysis was performed on the 12 items resulting from the EFA.

After deleting the atypical values (Tests for normality and outliers, AMOS), and having checked that they met the criteria of normality and linearity, three models were tested with the method of maximum likelihood: M1 one-factor, M2 two independent factors, and M3 two related factors (**Table 2**).

**Table 2:** Goodness-of-fit Indexes of the Proposed Models.

Models	χ^2^	CMIN/*df*	CFI	TLI	RMSEA	SRMR
(1) Factor	*p* < 0.001	187.456	0.782	0.762	0.124	0.149
(2) Independent factors	*p* < 0.001	2.568	0.863	0.833	0.099	0.214
(3) Related factors	*p* = 0.089	1.271	0.976	0.970	0.042	0.048

*CMIN, chi-square divided by degrees of freedom; CFI, comparative fit index; TLI, Tucker-Lewis index; RMSEA, root mean square error of approximation; SRMR, standardized root mean square residual.*

In the one-factor model and the two-independent-factor model, the value of the chi square was significant (*p* < 0.01), while the CFI, TLI and RMSEA fit indices did not reach optimal values. The model of two related factors was the only one with an adequate fit, a non-significant chi-square value, and CFI and TLI fit indexes with values higher than or equal to 0.970, and an RMSEA value lower than 0.05.

**FIGURE 1:**
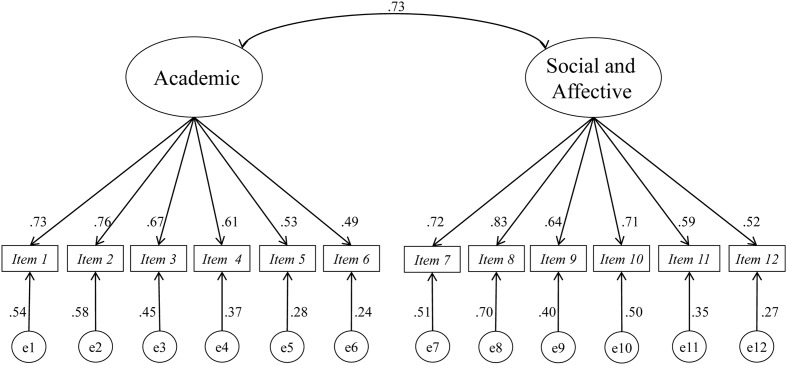
Two-related factor model of the “Questionnaire of attiudes toward learning teams” (CACTE).

The *t*-values (range 4.50–7.90) of the non-standardized regression coefficients were statistically significant. The standardized coefficients of Factor 1 ranged from 0.489, corresponding to Item 6 (“I learn more when working alone than on a team”), to 0.735, corresponding to Item 1 (“Working on a team increases my interest and motivation for the topics covered”). For Factor 2, they ranged from 0.517, corresponding to Item 12 (“Consensus among the team members helps to make the best decisions”), to 0.834 for Item 8 (“I feel comfortable working with my classmates on group activities”). The results of the model indicate that the two factors are related to each other (β = 0.730) (**Figure 1**).

**Table 3** shows evidence of the reliability of the questionnaire corresponding to the two-related-factor model, with values higher than 0.50 for AVE, and values of 0.80 for the CR and McDonald Omega coefficients.

**Table 3:** Values of AVE, CR and Ω of the CACTE scores.

	Total	Academic	Social and
	score	attitudes	affective attitudes
Average variance extracted	0.630	0.592	0.599
Composite reliability	0.952	0.895	0.897
McDonald’s Omega	0.934	0.841	0.870

In addition, to verify that the values obtained from the factor loadings are not conditioned by a single sample, the bootstrap method with a 95% confidence interval was applied. This method allows a large number of samples with data replacement to be created. Using a total of 1000 samples, **Table 4** shows that the values of the factor loadings are very similar to the values found in the CFA and that they are between the lower and upper limits of the 95% CI; therefore, all of them are significant (*p* ≤ 0.003).

**Table 4:** Bootstrap method, 1000 samples with a 95% Confidence Interval.

Factors	Items	Factor loadings	Mean 1000 samples	Lower limit	Upper limit	*p*
Factor 1 Academic attitudes	CACTE 1	0.735	0.734	0.544	0.861	0.003
	CACTE 2	0.762	0.762	0.640	0.856	0.002
	CACTE 3	0.674	0.675	0.502	0.787	0.002
	CACTE 4	0.610	0.601	0.466	0.728	0.002
	CACTE 5	0.530	0.529	0.415	0.700	0.003
	CACTE 6	0.489	0.484	0.401	0.658	0.003
Factor 2 Social and affective attitudes	CACTE 7	0.717	0.715	0.543	0.827	0.002
	CACTE 8	0.834	0.841	0.757	0.886	0.001
	CACTE 9	0.636	0.631	0.522	0.669	0.002
	CACTE 10	0.709	0.707	0.575	0.743	0.003
	CACTE 11	0.593	0.587	0.411	0.718	0.002
	CACTE 12	0.517	0.514	0.454	0.563	0.002

### Analysis of Gender Invariance

Next, a multi-group analysis was performed to determine whether the two-related-factor model is invariant by gender (115 females and 260 males). The comparison yielded no differences between the different models (*p* < 0.05) in the chi-square value and the ΔCFI values found in the unconstrained model, with differences of less than 0.01 in the CFI indexes between the four models, indicate that the factor loadings of the questionnaire are equivalent for women and men (**Table 5**).

**Table 5:** Multi-group Analysis of Gender Invariance.

Models	χ2	*df*	χ^2^/*df*	Δχ^2^	*p*	Δdf	CFI	TLI	SRMR	RMSEA
Model 1	143.89	106	1.358	–	–	–	0.950	0.938	0.055	0.042
Model 2	153.32	116	1.322	7,638	0.664	10	0.951	0.944	0.059	0.039
Model 3	153.51	119	1.290	8,162	0.833	13	0.954	0.950	0.059	0.037
Model 4	173.72	131	1.326	31,140	0.184	25	0.944	0.943	0.064	0.040

*Model 1 = Unconstrained; Model 2 = Measurement weights; Model 3 = Structural covariances; Model 4 = Measurement residuals*.

### Convergent Validity

Convergent validity refers to the degree to which the scores of an instrument correlate with those of another instrument that measures the same construct. In this case, in view of the difficulty in finding an instrument with sufficient evidence of construct validity and reliability that evaluates attitudes toward learning teams, and taking into account that the attitude toward teamwork determines willingness (or unwillingness) to work in a team (Gardner and Korth, 1998), it was decided to perform correlations and comparisons of means between the scores of the CACTE and the groups established as a function of the students’ preference to work alone or in a team, based on their response to “*I prefer to work alone or in a team*.” The findings showed that 47.7% claimed they prefer to work in teams (=1), 31% indicated that they were indifferent (=2), while 21.3% of the students preferred to work alone (=3). These response percentages were invariant by gender, χ^2^(2) = 0.180, *p* = 0.914, and grade, χ^2^(2) = 2.264, *p* = 0.322.

Regarding convergent validity, the Spearman correlation revealed the existence of significant inverse relations between preference for working alone and the total CACTE score (*r* = -0.437), academic attitudes (*r* = -0.568) and social and emotional attitudes (*r* = -0.464).

In addition, the ANOVA (**Table 6**) shows that the students who claimed to prefer working in teams obtained higher scores (*p* < 0.001) in the total score and in both factors of the CACTE. The pair comparisons with Bonferroni confirmed the existence of differences (*p* ≤ 0.003) between all the pairs compared.

**Table 6:** ANOVA CACTE/Groups’ preference for teamwork/working alone.

	1		2		3				
CACTE	*M*	*SD*	*M*	*SD*	*M*	*SD*	*F*	*p*	η^2^
Total	46.32	3.31	44.44	3.59	42.96	4.27	36.580	0.000	0.05
Academic attitudes	24.78	2.73	22.01	2.65	19.75	3.84	121.964	0.000	0.17
Social and affective attitudes	26.92	2.33	25.71	2.84	24.09	3.14	44.930	0.000	0.06

*1 = I prefer working in a team; 2 = I don’t care whether I work alone or in a team; 3 = I prefer working alone.*

### Nomological Validity

Nomological validity refers to the degree to which the relationships of a construct with other constructs, that form part of or an entire theory or theories, can be confirmed empirically (Wilson et al., 1989); i.e., whether the theoretical configuration of the data corresponds with the theoretical predictions of that configuration.

In this case, we related the CACTE scores with the factors of the *Learning Team Potency Questionnaire* (CPEA; León et al., 2017) and the *Academic Goals Questionnaire* (AGQ). We found medium/high significant correlations between the CPEA (**Table 7**) and the total score and the social and affective attitudes factor, and low correlations with the academic attitudes factor of the CACTE.

**Table 7:** Pearson Correlations between CPEA and CACTE Factors.

	CACTE Attitudes		
CPEA Team Potency	Total	Academic	Social and affective
Total	0.496^∗∗^	0.175^∗∗^	0.538^∗∗^
F1 Confidence	0.488^∗∗^	0.132^∗∗^	0.558^∗∗^
F2 Performance	0.420^∗∗^	0.193^∗∗^	0.424^∗∗^

*CPEA, Learning Team Potency Questionnaire; CACTE, Questionnaire of Attitudes toward Learning Teams. ^∗∗^The correlation is significant at the 0.01 level.*

With regard to the relation between academic goals and attitudes (**Table 8**), we found direct/low correlations between learning goals and the total score and both factors of the CACTE, and very low correlations between achievement goals and the total score and social and affective attitudes.

**Table 8:** Pearson Correlations between AGQ and CACTE Factors.

	CACTE		
AGQ	Total	Academic	Social and affective
Learning goals	0.295^∗∗^	0.221^∗∗^	0.288^∗∗^
Achievement goals	0.194^∗∗^	0.014	0.188^∗∗^
Social reinforcement goals	-0.060	-0.080	-0.082

*AGQ, Academic Goals Questionnaire; CACTE, Questionnaire of Attitudes toward Learning Teams. ^∗∗^The correlation is significant at the 0.01 level.*

Lastly, we conducted a multivariate analysis (ANOVA) to determine possible differences in the AGQ scores between students with more or less favorable attitudes toward teamwork. For this purpose, we divided the sample (*n* = 700) into three groups of the same size (33%) by means of a criterion of percentiles, assuming that the lower, middle and higher third of the total score of the CACTE correspond to subjects with unfavorable, favorable, and very favorable attitudes, respectively.

The ANOVA (**Table 9**) revealed the existence of differences in learning goals and achievement goals between the groups of attitude toward teamwork. The pair comparisons with Bonferroni confirmed that the differences between the unfavorable and very favorable pairs were significant.

**Table 9:** ANOVA AGQ Goals/Groups of Attitude toward Teamwork.

CACTE Attitudes toward teamwork									
	**Unfavorable**		**Favorable**		**Very favorable**				
**AGQ**	***M***	***SD***	***M***	***SD***	***M***	***SD***	***F***	***p***	**η^2^**
Learning goals	29.53	5.04	31.07	4.71	32.18	4.78	14.382	0.000	0.050
Achievement goals	26.81	3.27	26.83	3.15	27.61	3.22	3.715	0.025	0.014
Social reinforcement goals	9.77	3.27	9.76	3.47	9.39	3.00	0.813	0.444	0.003

*AGQ, Academic Goals Questionnaire; CACTE, Questionnaire of Attitudes toward Learning Teams.*

## Discussion

The aim of this study was to validate a questionnaire to measure attitudes to teamwork in higher education. The relevance of the study is based on the need to develop instruments that contribute to a better understanding and allow the evaluation and identification of the characteristics that promote a positive attitude toward teamwork, as a variable involved in the academic and social results of the team (Castelló, 1998). So their control is of great value, which justifies the idea of building viable and reliable instruments to evaluate a variable that, according to Lobato (1998), Johnson et al. (1999), Mena et al. (2013), and Mujika et al. (2013), are essential for teamwork.

The different analyses carried out confirm that the variables associated with the attitudes toward teamwork can be grouped into two solid, well-defined factors. According to Costello and Osborne (2005), factors with loadings greater than 0.50, made up of 4 or more items, are solid and of practical relevance.

In relation to the preference for teamwork, one in five (21.3%) participants in the present study prefers working alone, although most prefer to work in a team (47.7%). In this sense, the results of the different studies are very diverse. Nevertheless, Gottschall and García-Bayonas (2008), in a study with 1,249 university students of different degrees, found that more than one third prefer to work alone, with university students of Education being the ones who present the most positive attitudes toward teamwork.

In addition, the relations between the CACTE and the CPEA clearly indicate an association between expectations of team performance, confidence in classmates, and attitude toward teamwork, especially with regard to social and affective attitudes. Both (team potency and attitudes toward learning teams) motivational variables are related to group efficacy (Castelló, 1998; León et al., 2017).

Likewise, the analyses of the AGQ and the CACTE corroborated the relationship between goals and attitudes, suggesting that the participants with more favorable attitudes toward teamwork believe it helps them to reach their learning goals (learning, acquiring new knowledge and competences) and achievement goals (getting good grades and progressing in the studies). So teamwork is seen as a strategy and/or a resource (Valle et al., 2000) compatible with their learning and achievement goals.

On the other hand, although the CACTE presents sufficient evidence of validity and reliability, it is not exempt from limitations; such as the difficulty to generalize the results to other groups of university populations, which compromises the external validity (population and ecological) of the questionnaire, or to establish greater evidence of convergent and discriminant validity. As future lines of research, besides resolving these limitations, it would be of interest to validate the CACTE in non-university populations, as well as to examine whether attitudes toward teamwork are stable over time, whether the university changes these attitudes and in which direction, or whether teamwork methodologies, such as cooperative or collaborative learning, determine or are determined by attitudes.

## Conclusion

Lastly, based on the above, it can be concluded that the CACTE is a solid and robust instrument to measure attitudes toward learning teams, which can help to better understand their conceptual and empirical foundations. Its application is simple and fast, and it can be useful as a diagnostic and/or predictive measure, allowing us to know students’ attitudes toward teamwork in general or regarding a certain subject or material.

The CACTE has important implications for planning teaching and learning activities that contribute to improving the practice of teaching with respect to learning teams. It is our belief that teachers should create the conditions that can guarantee positive attitudes in learning teams. It is not sufficient to simply present the advantages of the effectiveness of teamwork to generate positive attitudes toward teamwork (Rudawska, 2017). Actually achieving it involves an effort and interest on their part, as well as the assumption that their role not only determines the correct functioning of the team and the achievement of the goals, but also the satisfaction and attitudes of all the students that participate in the different teams. Achieving the multiple advantages of group and teamwork methods in university classrooms requires careful programming on the teachers’ part; a programming that includes the design of activities incompatible with competition or individuality, interventions throughout the process to resolve conflicts, and an analysis of the teamwork that includes the students’ attitudes toward cooperation.

## Author Contributions

All authors listed, have made substantial, direct and intellectual contribution to the work, and approved it for publication. SML, BLB: analysis and interpretation of the data. SML, MPR, DIG, EFC, BLB: The conception and design of the work; Drafting the work.

## Conflict of Interest Statement

The authors declare that the research was conducted in the absence of any commercial or financial relationships that could be construed as a potential conflict of interest.

## References

[B1] Alford L. K. Fowler R. Sheffield S. (2014). Evolution of student attitudes toward teamwork in a project-based, team-based first-year introductory engineering course. *Paper Presented in 2014 at the ASEE Annual Conference,* Indianapolis, IN.

[B2] Allport G. W. (1935). “Attitudes,” in *Handbook of Social Psychology*, ed. Murchison C. M. (Worcester, MA: Clark University Press), 209–242.

[B3] Alonso-Tapia J. (2005). “Motivación para el aprendizaje: la perspectiva de los alumnos [Motivation for learning: the students’ perspective],” in *La Orientación Escolar en Centros Educativos [Academic Orientation in Schools]*, eds Otero A. Rivera P M.érez Solís (Madrid: MEC), 798–844.

[B4] Anderman L. H. Anderman E. M. (1999). Social predictors of changes in students’ achievement goal orientations. *Contemp. Educ. Psychol.* 25 21–37. 10.1006/ceps.1998.0978 9878206

[B5] Anderton-Lewis L. King T. (1995). An assessment of global communication awareness achieved through teamwork. *Delta Pi Epsilon J.* 39 12–23.

[B6] Beigi M. Shirmohammadi M. (2012). Attitudes toward teamwork: are Iranian university students ready for the workplace? *Team Perform. Manag. Int. J.* 18 295–311. 10.1108/13527591211251087

[B7] Burdett J. (2006). Degrees of separation – Balancing intervention and independence in group work. *Aust. Educ. Res.* 34 55–71. 10.1007/BF03216850

[B8] Castelló T. (1998). “Procesos de cooperación en el aula [Cooperation processes in the classroom],” in *Cooperar en la Escuela. La Responsabilidad de Educar para la Democracia [Cooperate in School. The Responsibility of Educating for Democracy]*, ed. Mir C. (Barcelona: Graó), 51–71.

[B9] Costello A. B. Osborne J. W. (2005). Best practices in exploratory factor analysis: four recommendations for getting the most from your analysis. *Pract. Assess. Res. Eval.* 10 1–9.

[B10] Ekimova V. Kokurin A. (2015). Students’ attitudes towards different team building methods. *Proc. Soc. Behav. Sci.* 186 847–855. 10.1016/j.sbspro.2015.04.157

[B11] Fransen J. Weinberger A. Kirschner P. A. (2013). Team effectiveness and team development in CSCL. *Educ. Psychol.* 48 9–24. 10.1080/00461520.2012.747947

[B12] Gardner B. Korth S. (1998). A framework for learning to work in teams. *J. Educ. Bus.* 74 28–33. 10.1080/08832329809601657

[B13] Gaudet A. D. Ramer L. M. Nakonechny J. Cragg J. J. Ramer M. S. (2010). Small-group learning in an upper-level university biology class enhances academic performance and student attitudes toward group work. *PLoS ONE* 5:e15821 10.1371/journal.pone.0015821 PMC3012112 21209910

[B14] Gottschall H. García-Bayonas M. (2008). Student attitudes towards group work among undergraduates in Business Administration, Education and Mathematics. *Educ. Res. Q.* 32 3–28.

[B15] Hall D. Buzwell S. (2012). The problem of free-riding in group projects: looking beyond social loafing as reason for non-contribution. *Active Learn. High. Educ.* 14 37–49. 10.1177/1469787412467123

[B16] Hammar Chiriac E. H. (2014). Group work as an incentive for learning–students’ experiences of group work. *Front. Psychol.* 5:558 10.3389/fpsyg.2014.00558 PMC4046684 24926282

[B17] Hayamizu T. Ito A. Yohiazaki K. (1989). Cognitive motivational process mediated by achievement goal tendencies. *Jpn. Res.* 31 179–189.

[B18] Hayamizu T. Weiner B. (1991). A test of Dweck ìs model of achievement goals as related to perceptions of ability. *J. Exp. Educ.* 59 904–915. 10.1080/00220973.1991.10806562

[B19] Hijzen D. Boekaerts M. Vedder P. (2006). The relationship between the quality of cooperative learning, students’ goal preferences, and perceptions of contextual factors in the classroom. *Scand. J. Psychol.* 47 9–21. 10.1111/j.1467-9450.2006.00488.x 16433658

[B20] Hijzen D. Boekaerts M. Vedder P. (2007). Exploring the links between students’ engagement in cooperative learning, their goal preferences and appraisals of instructional conditions in the classroom. *Learn. Instruct.* 17 673–687. 10.1016/j.learninstruc.2007.09.020

[B21] Ibarra M. S. Rodríguez G. (2007). El trabajo colaborativo en las aulas universitarias: reflexiones desde la autoevaluación [Collaborative work in the University classroom: reflections from self-assessment]. *Rev. Educ.* 344 355–375.

[B22] Järvelä S. Volet S. Järvenoja H. (2010). Research on motivation in collaborative learning: moving beyond the cognitive–situative divide and combining individual and social processes. *Educ. Psychol.* 45 15–27. 10.1080/00461520903433539

[B23] Johnson D. W. Johnson R. (2016). Cooperative learning and teaching citizenship in democracies. *Int. J. Educ. Res.* 76 162–177. 10.1016/j.ijer.2015.11.009

[B24] Johnson D. W. Johnson R. T. Holubec E. J. (1999). *El aprendizaje cooperativo en el aula.* Barcelona: Paidós.

[B25] Johnson D. W. Johnson R. T. Smith K. (2007). The state of cooperative learning in post-secondary and professional settings. *Educ. Psychol. Rev.* 19 15–29. 10.1007/s10648-006-9038-8

[B26] Kirschner F. Paas F. Kirschner P. A. Janssen J. (2011). Differential effects of problem-solving demands on individual and collaborative learning outcomes. *Learn. Instruct.* 21 587–599. 10.1016/j.learninstruc.2011.01.001

[B27] Krishen A. S. (2013). Catch it if you can: how contagious motivation improves group projects and course satisfaction. *J. Mark. Educ.* 35 220–230. 10.1177/0273475313495857

[B28] Lembke S. Wilson M. G. (1998). Putting the “team” into teamwork: alternative theoretical contributions for contemporary management practice. *Hum. Relations* 51 927–944. 10.1023/A:1016951611667

[B29] León B. Felipe M. E. Iglesias D. Latas C. (2011). Cooperative learning in the initial training of secondary school teachers. *Rev. Educ.* 354 715–729.

[B30] León B. Felipe E. Mendo S. Iglesias D. (2015). Habilidades sociales en equipos de aprendizaje cooperativo en el contexto universitario [Social skills in cooperative learning teams in the University context]. *Psicol. Conduct.* 23 191–214.

[B31] León B. Mendo S. Felipe E. Polo M. I. Fajardo F. (2017). Team potency and cooperative learning in the university setting. *J. Psychodidact.* 22 9–15. 10.1387/RevPsicodidact.14213

[B32] Lobato C. (1998). *El Trabajo en Grupo: Aprendizaje Cooperativo en Secundaria [Working in Groups: Cooperative Learning in Secondary Education].* Servicio Editorial. Leioa: Universidad del País Vasco.

[B33] McCorkle D. Reardon J. Alexander J. Kling N. Harris R. Iyer V. (1999). Undergraduate marketing students, group projects, and teamwork: the good, the bad, and the ugly. *J. Mark. Educ.* 21 106–117. 10.1177/0273475399212004

[B34] Mena I. B. Zappe S. E. Litzinger T. A. (2013). “Examining the experiences and perceptions of first-year engineering students,” in *Proceedings of the* *ASEE Annual Conference and Exposition,* Atlanta, GA.

[B35] Mendo S. León B. Felipe E. Polo M. I. Palacios V. (2016). Assessment of social skills of students of Social Education. *J. Psychodidact.* 21 139–156. 10.1387/RevPsicodidact.14031

[B36] Mujika M. G. Osinaga X. G. Uria E. S. Manso A. P. (2013). Developing teamwork efficacy factors: an experience in a project-based learning context. *Int. J. Eng. Educ.* 29 752–762.

[B37] Nausheen M. Alvi E. Munir S. Anwar R. (2013). Attitudes of postgraduate students towards cooperative learning. *J. Educ. Res.* 16 107–115.

[B38] Nokes-Malach T. J. Richey J. E. Gadgil S. (2015). When is it better to learn together? Insights from research on collaborative learning. *Educ. Psychol. Rev.* 27 645–656. 10.1007/s10648-015-9312-8

[B39] Payne B. K. Monk-Turner E. (2006). Students’ perceptions of group projects: the role of race, age, and slacking. *Coll. Stud. J.* 40 132–139.

[B40] Pfaff E. Huddleston P. (2003). Does it matter if I hate teamwork? What impacts student attitudes toward teamwork? *J. Mark. Educ.* 25 37–45. 10.1177/0273475302250571

[B41] Rodríguez F. J. Ridao S. (2014). El trabajo en equipo como recurso para fomentar las habilidades sociales en estudiantes universitarios [Teamwork as a resource to promote social skills in college students]. *Educ. Futuro Rev. Investig. Aplicada Exp. Educ.* 31 273–288.

[B42] Rudawska A. (2017). Students’ team project experiences and their attitudes towards teamwork. *J. Manag. Bus. Adm.* 25 78–97.

[B43] Ruiz Ulloa B. C. Adams S. G. (2004). Attitude toward teamwork and effective teaming. *Team Perform. Manag.* 10 145–151. 10.1108/13527590410569869

[B44] Serrano J. M. Calvo M. T. (1994). *Aprendizaje Cooperativo. Técnicas y Análisis Dimensional [Cooperative Learning. Dimensional Analysis and Techniques].* Murcia: Caja Murcia.

[B45] Ubillos S. Páez D. Mayordomo S. (2004). “Actitudes: definición y medición. Componentes de la actitud. Modelo de acción razonada y acción planificada [Attitudes: definition and measurement. Components of attitudes. Model of reasoned action and planned action],” in *Psicología Social, Cultura y Educación [Social Psychology, Culture, and Education]*, eds Fernández, I. Ubillos S. Zubieta E. Páez D. (Madrid: Pearson Educación), 301–326.

[B46] Urdan T. C. Maehr M. L. (1995). Beyond a two-goal theory of motivation and achievement: a case for social goals. *Rev. Educ. Res.* 65 213–243. 10.3102/00346543065003213

[B47] Valle A. González R. Núñez J. C. Suárez J. M. Piñeiro I. Rodríguez S. (2000). Enfoques de aprendizaje en estudiantes universitarios [Approaches to learning in students University]. *Psicothema* 12 368–375.

[B48] Vallejo P. M. (2006). *Medición de Actitudes en Psicología y Educación: Construcción de Escalas y Problemas Metodológicos [Measurement of Attitudes in Psychology and Education: Construction of Scales and Methodological Problems]* (Vol. 80) Madrid: Universidad Pontificia Comillas.

[B49] Venter I. Blignaut R. J. (1998). Teamwork: can it equip university science students with more rigid subject knowledge? *Comput. Educ.* 31 265–279. 10.1016/S0360-1315(98)00031-1

[B50] Wilson P. H. Spence S. H. Kavanagh D. J. (1989). *Cognitive-Behavioral Interviewing for Adult Disorders: A Practical Handbook.* London: Routledge.

